# Statistical Account of the Cases of Amputation Performed at the Pennsylvania Hospital from January 1, 1838, to January 1, 1840

**Published:** 1840-05-11

**Authors:** 


### BIBLIOGRAPHICAL NOTICE.

I.—*Statistical Account of the Cases of Amputation performed at the Pennsylvania Hospital from January* 1, 1838, *to January* 1, 1840. By G. W. Norris, M. D., one of the Surgeons to the Institution.

II.—*Statistics of the Amputations of Large Limbs that have been performed at the Massachusetts General Hospital; with Remarks.* By Geo. Hayward, M. D., one of the Surgeons to the Hospital.

Within the last few years, to the surprise of some of our surgical brethren, and amongst others to that of myself, it has been ascertained that the mortality after capital amputations was much larger than appeared at first sight. Many of us were under the impression that these operations were extremely insignificant, so far as the mortality was concerned. One of the editors of the Examiner laboured under this impression, and stated his convictions to some of his surgical friends at Paris; after his return to America, he found that the amputations at the Pennsylvania hospital were very often fatal; that is, during a portion of the period alluded to by Dr. Norris as that of greatest mortality after amputations, (1834–6.) Dr. Norris was also misled, trusting to his recollections of the mortality during a short period. On his return to America he has examined the records of the cases at the Pennsylvania hospital during a period of ten years, and finds that the mortality was very considerable, but varied at different parts of the ten years, being at times very small and at others very great.

At Boston, the mortality has, on the whole, been less considerable, but still is much greater than would have been believed by many who were not accustomed to look to accurate statistics as their guide.

The following extracts from the two papers will give the important facts contained in them.

We begin with Dr. Norris:

"Of eighty amputations on seventy-nine patients, performed during a term of ten years at the Pennsylvania Hospital, thirty-five were primary, of which twenty-four were cured, and eleven died, four of the deaths occurring within the twenty-four hours immediately following it.

Twenty were secondary, of which thirteen were cured and seven died.

Twenty-five* were for the cure of chronic affections, of which twenty were cured and four died.

Thirty-two of the amputations were of the upper extremity, of which twenty-seven were cured and five died.

Forty-seven were of the lower extremity, of which thirty-one were cured and sixteen died.

Seven were amputations at the joints, of which four were cured and three died.

Of the seventy-nine operated on,

| | | | |
|---|---|---|---|
| 13 were under 20 years, | of whom | 12 were cured, | 1 died. |
| 26 were between 20 and 30, | of whom | 19 were cured, | 7 died. |
| 22 were between 30 and 40, | of whom | 15 were cured, | 7 died. |
| 16 were between 40 and 50, | of whom | 9 were cured, | 7 died. |
| 2 were upwards of 50, | of whom | 2 were cured. | |
| 79 | | 57 | 22 |

The conclusions to be drawn from an analysis of the two tables which I have now published are,

1. That amputation† with us is to be regarded as an operation attended with much danger to the life of the individual, the mortality after it being 1 in 3 7-11ths.

2. That the chances of success after it are much greater in persons who have been for some time suffering from chronic diseases, than in those who have it done while enjoying robust health, the mortality in the former class of cases being 1 in 6¼, while in the latter it is 1 in 3 2-11ths.

3. That immediate amputations after injuries

---

* One double.

† The great amputations only, it will be recollected, are alluded to. No death has followed any of the amputations of fingers, or toes, which have been made in the hospital during the ten years past.

are less fatal than secondary operations, the mortality after the former being 1 in 3 2-11ths, while in the latter it is 1 in 2 6-7ths.

4. That amputation of the lower extremity is much more fatal than that of the superior member, the mortality after the former being 1 in 2 15-16ths, while in the last mentioned class of cases it is only 1 in 6 2-5ths, and

5. That the danger increases with the age of the individual operated on."

Dr. Hayward agrees in most respects with the conclusion of Dr. Norris. His remarks are:

From this table it appears, that there were seventy operations on sixty-seven patients; three patients having two limbs removed. In one of these three cases, one operation was above and the other below the knee, and in the other two, both operations were below; the first patient died, and the other two did well.

Of the whole number operated on, fifteen died and the remainder recovered, at least so far as to be able to leave the hospital; though it is probable that in some instances the disease may have returned.

There were thirty-four patients who had the thigh amputated, and one of these had the other leg taken off at the same time below the knee; of this number, nine died. Of twenty-three patients whose legs were amputated below the knee, two having both legs removed, five died; and of the ten who had an arm amputated, six below and four above the elbow; one died.

This goes to confirm the prevailing opinion among surgeons, that amputation of the lower extremities is more often followed by fatal consequences than that of the upper, and that death takes place more frequently after amputation of the thigh, than after that of the leg. More than a quarter of those whose thighs were amputated died, while there was but little more than one death in five among those whose legs were removed below the knee, and only one of the ten whose arms were amputated. This patient too died of delirium tremens. The operation to be sure did not arrest the disease, but apparently contributed nothing to the fatal result.

This table tends also to support the opinion, that patients who undergo amputation for chronic diseases are much more likely to recover than those in whom it is performed in consequence of recent accidents. Of the first class, there were forty-five patients afflicted with various diseases, and of this number all recovered but six; and of the remaining twenty-two, whose limbs were removed on account of recent injuries, no less than ten died; being nearly half of the latter and less than one in seven in the former.

This fact certainly gives support to the opinion, that a state of high health is not favourable to surgical operations; and it also tends to show that death after amputation is not by any means attributable in all cases to the operation alone; for if it were, the proportion of deaths should be as large among one class of patients as among the other. There can be no doubt, I think, that the result is influenced very much not only by the age and constitution of the patient and the disease or injury for which the operation is performed, but also by the period at which it is done. I have before said that I thought that amputation was 'often performed when it might have been avoided.' But this remark applies principally to cases of recent injury. In those of chronic diseases of the limbs, the error is more apt to be of the opposite character; the operation is either not performed, or if done at all, frequently not till it is too late. It cannot be denied, I think, that there is a disposition at the present day to defer amputation too long in cases of diseased limbs; there is an unwillingness to admit that the morbid affection is beyond the reach of remedies, and the operation is too often postponed till other parts become affected, or the system is worn down by continued irritation. At length the limb is removed; but the patient, already exhausted by disease and long suffering, is hurried to his end by the very means that might have saved him, if they had been earlier employed.

If amputation is frequently too long delayed in chronic diseases of the limb, it is, I fear, very often resorted to in recent injuries earlier than it should be. Many limbs that have been removed, might probably have been saved; but where this cannot be done, it is rare that much inconvenience would follow from a little delay.

In most cases of accident sufficiently severe to justify amputation, the whole system has suffered a great shock, and an operation at this time, before reaction is fairly established, is very likely to cut off what little chance the patient might otherwise have of recovery. While the extremities are cold and the action of the heart is feeble, the local injury is hardly, if at all, perceived, and adds nothing to the patient's sufferings. An operation cannot be required then; and yet how often it is done at that period; the better judgment of the surgical attendant sometimes being overruled by the importunate interference of the bystanders.

If the injury be not so serious as to cause almost immediate death, reaction usually comes on with proper management in a few hours, and then, if an operation be necessary, it can be done with a much greater prospect of success.

With regard to the ages of the patients operated on, it appears that there were

| | | | | |
|---|---|---|---|---|
| Under 20 | years | 13, | of this number | 1 died. |
| Over 20, not exceeding 30 | " | 31, | " | 8 " |
| " 30, " 40 | " | 9, | " | 3 " |
| " 40, " 50 | " | 10, | " | 2 " |
| " 50, " 60 | " | 3, | " | 1 " |
| Over 70 | " | 1, | " | 0 " |

Whole number, 67. No. of deaths, 15.

*Am. Journ. Med. Science.*

The combined results are as follows:

Of 124 cases of amputation, 37 proved fatal, or a fraction more than 1 in 4. This mortality of one-fourth in the amputations in the two hospitals which are perhaps placed upon a better footing than any others in America, shows the great mortality, whether this results from the operation or from the cause which rendered it necessary. The results of Mr. Benjamin Phillips are founded on a collection of 640 cases, and give a mortality of 1 in 4 4-15ths, a little less than that of the American statistics, but probably not differing from it if the calculation were based on a larger number of cases. As we shall presently show, the excessive mortality at Paris does not follow amputations at all the hospitals, but is confined to those most unfavourably situated.

These statistical accounts are of great value, as exhibiting the gravity of an operation, the dangers of which have been so generally underrated, and we trust that they will be continued, as from the variableness of the results during the different years, we think they should embrace a still longer period, before we can decide, with sufficient accuracy, upon the average mortality in our own hospitals; and that they should be compared with similar statistics from European hospitals before venturing to claim for ourselves a greater proportional success. Thus, Dr. Hayward mentions, "It has been stated that more than one-half of all whose limbs are amputated at some of the hospitals of Paris, die." This statement is probably not founded on regular statistics, and, although we do not doubt its correctness in the case of a single hospital, (the Hotel Dieu,) yet there are other hospitals that occasionally offer a brilliant contrast to this sad picture, and, by their success, diminish the average mortality many-fold.

Thus, during the year ending August, 1837, there occurred in the service of Velpeau, at "la Charité," eleven amputations of limbs, distributed as follows: Of thigh, three; of leg, four; of foot, one; of arm one; of forearm, two; of these ten recovered, and only one, an amputation of the leg in the articulation, died. But as this remarkable degree of success would not be admitted as the basis of a comparison between the results obtained in the two countries, so neither should the frequent failures in one or more of the unhealthy Parisian hospitals, be assumed as giving the average mortality in that city. Statistics from all their hospitals, for a number of years, must be compared with our own, before we can arrive at truth; and, we believe, could the Hotel Dieu be excluded from the account, that the difference in success would not be so much in our favour as is generally supposed.

But, valuable as these statistics are in their present form, we conceive that their value would be infinitely enhanced by a few additional details. They prove, beyond a doubt, the great risk to life attendant upon the operation, a risk not hitherto appreciated. They demonstrate, by a rigid calculation of numbers, that, of all those subjected to amputation of a limb, more than one quarter die. But an acquaintance with this fact is but of moderate *practical* importance to the surgeon (except as inducing him to refrain from amputations of "complaisance,") so long as it is unaccompanied by a knowledge of the immediate causes which lead to a fatal termination.

Amputation cannot be proscribed as an operation, nor can its frequency be even greatly diminished. Circumstances will continually render it indispensable, whatever may be its dangers, and hence the great importance of examining upon what its dangers depend, of learning, in a word, what is the common cause of death after a great amputation. We have been led to ascribe it, in a large majority of the fatal cases, to a purulent resorption, or a purulent phlebitis, in which the veins of the medullary membrane are the efficient agents. Thus, out of thirteen great amputations occurring during a year in the hospital of St. Louis, five were cured and eight died; of these, six were examined after death, and five out of the six presented metastatic abscesses of the viscera; the sixth died of hospital gangrene. Subsequent examinations in this city tend to confirm the opinion, that a large majority of those who die after amputation of a limb, die from a chemical decomposition of the blood consequent upon the admixture of purulent or gangrenous matter with this fluid. But statistics alone can settle this point. How important, then, to append to the valuable statistics already furnished, the causes of death in the fatal cases, whenever they can be procured. For in a knowledge of these causes consists the first step towards a rational plan for diminishing the amount of mortality.

In connexion with this subject, we may advert to the fact, that without ever appearing as an actual epidemic, metastatic abscesses are, in some years, a more frequent consequence of amputation than in others, and appear to be somewhat under the control of atmospheric or other influence; thus probably, " cœteris paribus," accounting for the variableness of success in different years, in confirmation of which we may mention, that the late success in amputations at the Pennsylvania Hospital, coincides with a similar happy termination of compound fractures of the thigh, which so generally prove fatal, and almost constantly from this cause.

Another important addition to the statistics as they are now offered, would be the mention of the point at which the bone was sawed, in order that we might judge whether the nutritious artery was severed in its canal, and the medullary membrane thus deprived of its supply of blood, and what influence this section of the artery might have upon the subsequent necrosis of the bone. The great frequency of this necrosis in the Parisian hospitals is notorious. M. Reynaud states, that during two years, in the service of M. Roux, every amputation of the thigh, without exception, died, and in almost every instance he was able to recognise this affection of the bone of the stump. He, together with Serre of Montpelier, ascribes it to the action of the saw on the medulla, producing an inflammation which, being imprisoned, causes strangulation of the vessels and the death of the bone; the latter asserting that, during three years that he has employed a saw with fine teeth, the accident has never occurred. To statistics, again, we must look for a correct decision upon this subject.

But the point at which the amputation is performed in the leg, has, at the present moment, an importance of an entirely different character. Since the introduction of the artificial leg of M. Martin, which offers a very perfect substitute for almost all the natural motions of the foot without pressing upon the stump, but requires that this stump should be sufficiently long to act as a lever, it becomes the duty of the profession to decide upon the comparative dangers of an amputation at different points of the limb; and if, as it would appear, the dangers of amputation are absolutely less in proportion as we approach the ankle, it must lead to the establishment of a law, entirely subversive of the practice now generally pursued, viz., in an ablation of the lower extremity, to amputate as near the ankle as circumstances will admit. Upon the comparative dangers of the operation in different points of the limb, statistics alone can enlighten us, and we are induced to hope that those who have already done so much for the profession, by exhibiting the serious character of a great amputation in its true light, will go still farther, and elucidate those points upon which the gravity of the operation depends. W. P. J.

